# Machine Learning Techniques Accurately Classify Microbial Communities by Bacterial Vaginosis Characteristics

**DOI:** 10.1371/journal.pone.0087830

**Published:** 2014-02-03

**Authors:** Daniel Beck, James A. Foster

**Affiliations:** Department of Biological Sciences and Institute for Bioinformatics and Evolutionary Studies, University of Idaho, Moscow, Idaho, United States of America; University of Illinois, United States of America

## Abstract

Microbial communities are important to human health. Bacterial vaginosis (BV) is a disease associated with the vagina microbiome. While the causes of BV are unknown, the microbial community in the vagina appears to play a role. We use three different machine-learning techniques to classify microbial communities into BV categories. These three techniques include genetic programming (GP), random forests (RF), and logistic regression (LR). We evaluate the classification accuracy of each of these techniques on two different datasets. We then deconstruct the classification models to identify important features of the microbial community. We found that the classification models produced by the machine learning techniques obtained accuracies above 90% for Nugent score BV and above 80% for Amsel criteria BV. While the classification models identify largely different sets of important features, the shared features often agree with past research.

## Introduction

### Microbial Communities and Disease

Microbial communities play critical roles in human health and disease. For example, gut microbial communities have been linked to obesity [1], [2], lung communities to pulmonary infections [3], and vaginal communities to bacterial vaginosis [4]–[6]. The complexity of these communities, however, makes determining specific causes of disease difficult.

In many natural environments, next-generation 16S rRNA sequencing unveils hundreds to thousands of microbe types. Everything from the physiology to the ecological roles of most of these microbes remains unknown. These microbes are difficult to study, both due to their large numbers and our inability to culture many of them in the lab [7]. The composition of these communities may fluctuate widely with environmental factors or as a result of microbial interactions.

### Vaginal Microbiome and Bacterial Vaginosis

The vagina microbiome is complex, with microbial composition varying between women and over time. This variation may be caused by immune factors, environmental variables, or dynamic microbial interactions. In some women the microbial community includes hundreds of microbe types, while in other women, the microbial community is dominated by a single species, often in the *Lactobacillus* genus [8]–[10]. Across women, the communities appear to cluster into distinct community types.

Bacterial vaginosis (BV) is a common condition, affecting up to 29% of all women [11]. BV is associated with increased risk for some STDs and preterm birth. Researchers have defined BV in two common ways. In clinical settings, Amsel criteria are often used. Amsel criteria include the presence of discharge, a positive whiff test, the presence of clue cells, and a pH greater than 4.5. Amsel criteria BV is defined by the presence of at least three of these criteria [12]. Nugent score is a second way to define BV. The Nugent score relies primarily on counting gram-positive cells with morphologies similar to some *Lactobacillus* sp. (large rods) [13]. Nugent scores range from 0 to 10, with BV defined as a score greater than or equal to 7. The two definitions for BV lead to some interesting results. Using Nugent score BV definitions, up to 30% of all BV diagnoses are “asymptomatic”, meaning that the woman in question has no symptoms though her microbiome elicits a high Nugent score, perhaps because her “normal” microbiome happens to contain more species with large rods than most other women. The significance of this phenomenon is uncertain.

It is difficult to identify a single cause of BV, even though the microbial community and BV are correlated. The number of microbe types found within the vagina microbiome is very large and the number of possible interactions between these microbes is even larger. In addition, noise in the data may obscure relationships between the microbial community and BV. Different bacterial consortia may also provide very similar functionality.

### Machine Learning and Models

These difficulties are analogous to a problem faced by genetic epistasis researchers, where there are so many possible genetic interactions that may be linked to disease that it is difficult to determine the few that really matter. In this study, we applied three machine learning algorithms that have successfully discovered genetic interactions associated with disease to uncover possible microbial interactions associated with BV. In particular, we build models of BV diagnosis in the form of classifiers that were discovered with genetic programming (GP) [14], [15], random forests (RF) [16], [17], and logistic regression (LR) [17].

Genetic programming uses computational analogs of evolutionary processes to search for highly fit models. In our case, these models are decision trees where the leaves are features that may be relevant to diagnosing BV, and where internal nodes are functions that operate on data passed on from their dependent nodes. GP transforms a population of candidate models by combining substructures from multiple “parent” models, modifying individual models randomly, and retaining only those models that are better at classifying BV from our input datasets for the next iteration. When the algorithm is stopped, the best model in the final population tends to be a very good predictor of BV. To determine which microbial populations or patient behaviors were most closely associated with BV, we analyzed which features were in the best GP classifiers and how they were used.

GP is very flexible and allows nearly unlimited model complexity. However, it searches for models stochastically and does not exhaustively search all possible models. In addition, the models produced by GP can be very large, and are often difficult to interpret. Also, computation costs tend to be high.

Random forests is an ensemble technique that builds a population of tree classifiers, where the final classification of a given set of features is its most frequent classification by the team members. RF is computationally efficient but may not be as flexible as GP. It is easier to extract important model features from RF models than from GP models, but not as easy as with logistic regression.

Logistic regression fits a linear model to the data, producing a linear combination of features and regression coefficients whose value for a given set of microbial communities and patient behaviors (in our case) quantifies the likelihood that the patient had BV. There are many ways to build the LR model. We use a maximum likelihood method implemented in the R package *glmnet* [18], in which the final model was parameterized in such as way as to maximize the probability that this set of features was associated with BV. Features were selected for inclusion in the model by *glmnet* using the lasso [19]. It was then straightforward to determine which features were most useful in BV diagnosis: the magnitudes of the regression coefficients indicate the weight given to the corresponding feature.

LR is computationally very efficient. And the fitted model is easy to interpret. However, the structure of the final model is dependent on how terms are added to the regression equation, and LR may not be appropriate for non-linear phenomena. LR models are the easiest to interpret of the three in this study.

### BV Diagnosis as a Classification Problem

In this paper we apply these machine learning methods to classifying microbial communities into BV+ and BV− categories. We show that the methods accurately classify women by BV status based on their vagina microbiome and associated environmental factors. Additionally, we identify the parts of the microbial community that seem to play important roles in determining BV status.

We are interested in two aspects of the classification models, classification accuracy and feature usage. The accuracy of the models is a measure of how well they partition samples into diseased and non-diseased categories. We measure accuracy as the percentage of correctly classified samples. Different machine learning algorithms have different ways of selecting and weighting features, so our analysis of feature usage was algorithm specific.

## Results

**Figure 1:**
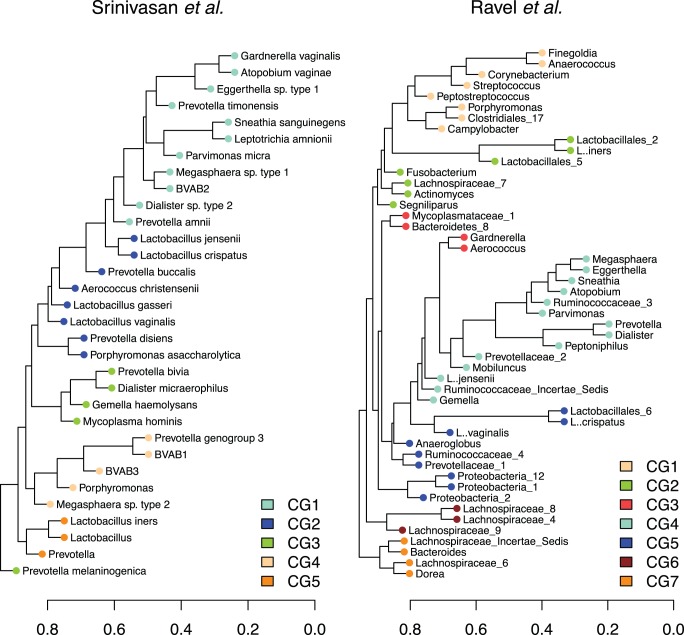
The correlated microbe groups. This figure shows the correlated microbe groups. We converted the *sparCC* correlations between microbial taxa to distances by subtracting the absolute value of the correlation from one. We then clustered the taxa and defined correlated groups using a dynamic tree-pruning algorithm (from the R library *dynamicTreeCut*). Microbial taxa not falling into these groups are not shown.

Before generating classification models, we first collapsed many of the microbes into groups based on correlations. We did this to both reduce the number of factors and to increase the interpretability of the classification models. The groups of correlated microbes are shown in Figure 1. We used two different datasets to train and evaluate the models, one from Srinivasan *et al.* [9] and one from Ravel *et al.* [8]. The two datasets produced different correlated microbe groups. There is some similarity in the groups, for example CG1 in the Srinivasan *et al*. dataset shares many microbes with CG4 in the Ravel *et al.* dataset.

**Figure 2:**
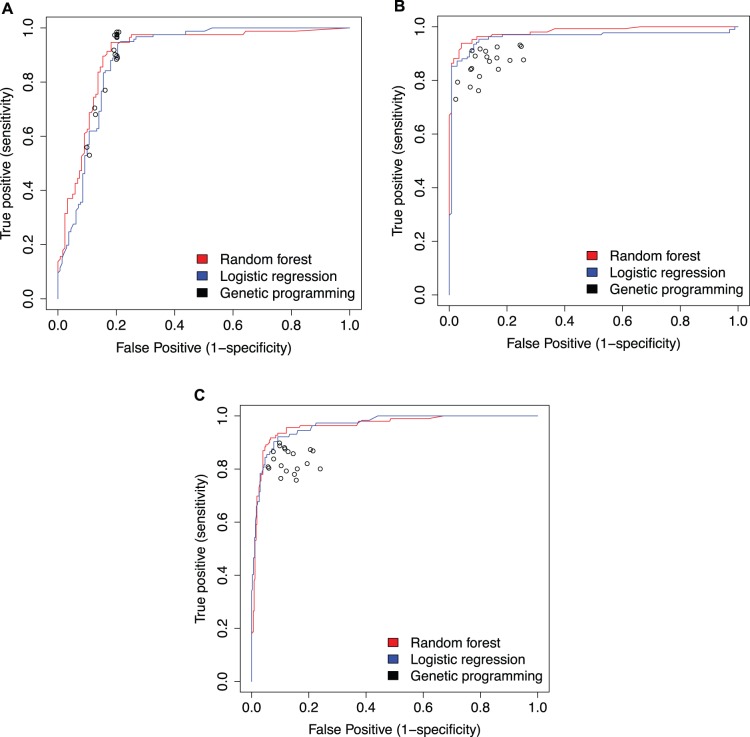
A comparison of the classification accuracies for each machine learning technique. This figures shows the accuracy of different classifiers at classifying microbial communities into BV categories. The red and blue lines show the accuracy of random forest and logistic regression classifiers respectively. The black dots are different genetic programming models. Panel A shows the results using the Srinivasan *et al.* dataset and Amsel BV. Panel B uses the Srinivasan *et al.* dataset and Nugent score BV. Panel C uses the Ravel *et al.* dataset and Nugent score BV.

After obtaining classification models using GP, LR, and RF, we evaluated the accuracy of the models with receiver operator curves (ROCs). ROCs show the performance of the model at classifying both BV+ and BV− samples. This allows us to simultaneously compare the type 1 and type 2 errors for each model. Figure 2 shows the ROCs for each of the analyses. A perfect model would have a curve that forms a right angle in the upper left of the ROC. More accurate models have a true positive rate closer to 1 and a false positive rate closer to 0.

As can be seen in Figure 2, both LR and RF tend to outperform GP. However, the accuracy of all the machine-learning techniques was remarkably similar. RF and LR models obtained accuracies consistently between 90% and 95% when classifying on Nugent score BV. GP models often classified samples with similar accuracies, but high variation between GP models reduced the average GP accuracy. The models perform slightly worse when classifying on Amsel criteria BV. However, all three techniques obtained accuracies above 80%.

After determining the accuracy of the models, we deconstructed the models to determine which features were most useful. We ranked the features by their apparent importance to each model. The top fifteen features for each classification technique are shown in Table 1. There is little overlap between the important factors used by the classification models. For the Srinivasan *et al*. dataset, when classifying by Amsel criteria BV, the Nugent score is the only important feature shared by all classification techniques. Four other features are shared between GP and either RF or LR. The results are similar when classifying by Nugent score BV. For the Srinivasan *et al.* dataset, CG2 and the whiff test results are identified by all three techniques. For the Ravel *et al*. dataset, all techniques identify CG4.

**Table 1:** This table shows the fifteen most important features identified by the different classifiers.

Srinivasan *et al.* - Amsel			Srinivasan *et al.* - Nugent score			Ravel *et al.* - Nugent score		
Genetic Programming	Random Forests	Logistic Regression	Genetic Programming	Random Forests	Logistic Regression	Genetic Programming	Random Forests	Logistic Regression
nugent^a^	nugent^a^	Prevotella genogroup 7	pH^b^	CG2^a^	clue^b^	CG4^a^	CG4^a^	CG1^b^
Moryella indoligenes	CG1	Streptococcus agalactiae^b^	CG2^a^	CG1^b^	CG1^b^	pH^b^	pH^b^	Lactobacillus 2
Mycoplasma^b^	Uncorrelated microbes	Peptoniphilus lacrimalis	whiff^a^	CG5	race	GpI	CG3	Sutterella
Fusobacterium	CG3	Bifidobacteriaceae	Sutterella wadsworthensis	vag_fluid	whiff^a^	Coriobacteriaceae 3	Ureaplasma	CG6
Sutterella wadsworthensis	CG4	Raoultella planticola	Neisseria gonorrhoeae	pH^b^	Finegoldia magna^b^	Peptostreptococcaceae Incertae Sedis	CG5	Total number reads^b^
Bacteroides Porphyromonas^b^	Anaerococcus prevotii tetradius	Mycoplasma^b^	Bacteroides	whiff^a^	CG2^a^	Flexibacteraceae 5	Total number reads^b^	Bulleidia
CG5^b^	CG2	nugent^a^	Haemophilus	clue^b^	Streptococcus anginosus	Moryella	CG2^b^	Proteobacteria 3
Streptococcus agalactiae^b^	CG5^b^	Peptoniphilus harei	Bacteroides xylanisolvens	CG4	Peptostreptococcus	Megamonas	CG1^b^	Bilophila
Veillonella montpellierensis	Peptostrepto-coccus	Dialister propionicifaciens	Eubacteriaceae Lachnospiraceae	Streptococcus agalactiae^b^	Streptococcus agalactiae^b^	Enterobacteriaceae 2	Ethnic Group	CG2^b^
Candidatus Peptoniphilus massiliensis	Arcanobacterium phocae	Fusobacteriaceae	Arcanobacterium phocae	CG3	Raoultella planticola	Chryseobacterium	Uncorrelated microbes	Lactobacillales 1^b^
Clostridiales	race	Megasphaera micronuciformis	Eubacteriaceae Ruminococcaceae	Finegoldia magna^b^	Streptococcus parasanguinis	Patulibacter	Lactobacillus gasseri	CG4^a^
Bifidobacterium breve	Coriobacteriaceae	Porphyromonas sp. type 1	Mobiluncus curtisii	Uncorrelated microbes	Megasphaera	Haemophilus	Clostridiales 15	Salmonella
Haemophilus	Actinomyces	Haemophilus pittmaniae	Campylobacter ureolyticus	Lactobacillus coleohominis	Prevotella genogroup 4	Clostridia 2	Community group	Dermabacter
Streptococcus salivarius thermophilus	Bacteroides Porphyromonas^b^	Neisseria gonorrhoeae	Delftia tsuruhatensis	Anaerococcus vaginalis	Streptococcus salivarius thermophilus	Rothia	Lactobacillales 1^b^	Flexibacteraceae 2
Fusobacterium periodonticum	Finegoldia magna	Asticcacaulis excentricus	Ruminococcaceae	Streptococcus mitis oralis	Pseudomonadaceae	Bacillus c	Staphylococcus	Exiguobacterium

Features common to all three techniques are labeled ‘a’. Features common to two techniques are labeled ‘b’. The features are listed in order of importance.

The different classification techniques varied widely in computational time. LR and RF were relatively quick, usually completing in less than an hour on a single laptop. GP, on the other hand, took several hours longer.

## Discussion

This study demonstrates the feasibility of using classification models to identify important microbial community features related to BV. However, the results of this study also show many complications that must be taken into account when designing future studies.

First, we can look at the results of the classification techniques within a single dataset. Classifier accuracy is similar between the three techniques. The accuracy obtained by each classification method is high, often exceeding 80% regardless of the dataset or classification technique. The strength of the classification accuracy indicates the presence of some signal of BV in the dataset. A better than random classification accuracy indicates the presence of some feature in the dataset that is associated with BV.

The GP results show wider variation between models when the classification phenotype is Nugent score BV. This variation is not seen when classifying based on Amsel criteria BV. While the cause of this variation is unclear, there are a number of possible explanations. GP can theoretically explore a much larger set of possible models than RF and LR. This wider exploration, in combination with a large stochastic component, may increase the variation in the GP model accuracy. Additionally, the specific GP implementation we use may not efficiently avoid local optima. Further optimization of GP methods may increase overall accuracy and decrease its variation between models.

The high accuracy of the classification techniques indicates the presence of some association between some dataset features and BV. The top fifteen important features for each technique are shown in Table 1. These results are interesting for many reasons. The few features that overlap differ between the three analyses. In the Srinivasan *et al.* dataset, when classifying on Amsel criteria BV, Nugent score is the only important feature shared by all classification techniques. When classifying on Nugent score BV, the whiff test and CG2 were important to all three techniques. Similarly, the Ravel *et al.* dataset resulted in CG4 and pH selected by all three techniques. These factors have often been identified by previous studies as correlates with BV [4], [5]. BV defined by Nugent score overlaps with BV defined by Amsel criteria. This may explain the apparent importance of Nugent score when classifying by Amsel criteria BV. Similarly, the presence of Amsel criteria such as vaginal discharge and odor, clue cells, and pH when classifying by Nugent score BV likely reflects the overlap between Amsel criteria BV and Nugent score BV. Ravel *et al*. identified a group of microbes that overlaps substantially with CG4, which all three techniques identified as important. This group includes *Megasphaera*, *Eggerthella*, *Sneathia*, *Prevotella*, and *Dialister*, among others.

While the important features identified by all classification techniques seem to agree with previous research, there are many features that are shared by only two techniques, or are unique to a single technique. In fact, the majority of the first fifteen important features are unique to a single classification technique. This lack of overlap has many possible explanations. Using the first fifteen most important features identified by each technique is an arbitrary choice. Our analysis doesn’t determine how each feature affects the overall classification accuracy. Additionally, the use of one feature may change the relative importance of the remaining features. This may amplify small differences in the classification techniques, resulting in very different sets of important features.

Our analysis highlights an important aspect of using classification models to detect parts of the microbial community that are associated with BV. The features included in the analysis are likely important to the outcome. This can be seen in the important features identified by the techniques. Amsel criteria are found to be important when classifying by Nugent score BV and Nugent score is identified as important when classifying by Amsel criteria BV. These findings may be unsurprising, as both the Amsel criteria and Nugent score attempt to diagnose BV. It may be more informative to remove these features from the dataset before applying the classification techniques.

While each technique obtained similar classification accuracy, technical characteristics of the techniques differentiate them in important ways. A key consideration for these techniques is the easy extraction of important features. This is a difficult problem for large and complex GP models. The approach we take in this study is to determine how varying the value of each feature independently affects the overall model accuracy. Additionally, we count the number of replicate GP models that include the feature. We combined these two measures to produce an overall importance measure for each feature. However, it is unknown whether this is optimal. We may be missing important parts of the GP models. This problem is somewhat alleviated for RF and LR models. Extensions to this study may include using machine learning techniques designed for easy identification of important features. Computational time may also be important to some researchers. The RF and LR analyses were relatively quick, completing in less than an hour on a single computer. The GP analysis, however, took several hours longer.

While we applied these classification techniques to two different datasets, these results are not comparable for a variety of reasons. Similar considerations will often apply to comparisons of techniques for classification-based diagnostics using multiple datasets. First, the types of samples collected in the two studies differed. The Srinivasan *et al*. study included women with and without a BV diagnosis. The Ravel *et al*. study included only asymptomatic participants. While both studies use Roche’s 454 FLX sequencing platform, they amplify different regions of the 16S rRNA sequence. Srinivasan *et al*. use the V3–V4 region while Ravel *et al*. use the V1–V2 region. Additionally, the studies use different methods for classifying reads into taxonomic groups (the *RDP classifier* [20] in the Ravel *et al*. study and *pplacer* [21] in the Srinivasan *et al*. study).

In our study, we analyzed the results for each study individually, using the same read identification used in the original study. This allowed us to compare our results with the previous ones. However, this approach has the consequence of making it difficult to compare the results for the two datasets. This difficulty is shown clearly in the identification of different correlated groups. The correlated groups often include different microbial taxa. An additional difficulty is the comparison of a species level identification in the Srinivasan *et al*. study with genus level identification in the Ravel *et al*. study.

In spite of these dataset differences, a few patterns in the results may motivate future work. The ROC plots in Figure 2 show accuracies for the Nugent score BV classifiers that are remarkably similar between datasets. It seems possible that this similarity reflects a consistent property of the dataset. Application of these classification techniques on different microbial community phenotypes (such as obesity or pH) may determine if these patterns are significant.

In this study we have shown that GP, RF, and LR generate models that classify samples by Amsel criteria BV with accuracies above 80%. These same techniques classify samples by Nugent score BV with accuracies above 90%. This study demonstrates the feasibility of using classification models to identify populations in a microbial community that are associated with BV. Determining the effect size of the important features may extend these results. Additionally, applying these techniques to different datasets and classifying on a variety of microbial community characteristics will determine how well these methods work for samples that may be very different from the vagina microbiome.

## Materials and Methods

### Dataset Details

We use two different datasets drawn from studies published by Ravel et al. in 2011 [8] and Srinivasan et al. in 2012 [9]. The Ravel et al. study sampled the microbiome of 396 asymptomatic women. The study amplified and sequenced the V1–V2 variable regions of the 16S rRNA gene using Roche’s 454 FLX sequencer. Reads were classified at the genus level using the RDP classifier [20]. The reads identified as Lactobacillus were further classified to the species level using a hidden Markov model based algorithm. The study identified a total of 282 microbial taxa across all samples. Out of 396 samples, 97 were BV+ using a Nugent score definition.

The Srinivasan *et al*. study sampled the microbiome of 220 women, 97 of whom were BV+ using Amsel criteria BV. Similarly, using Nugent score BV, 117 women were BV+. The study amplified and sequenced the V3–V4 variable regions of the 16S rRNA gene using Roche’s 454 FLX sequencer. Reads were classified at the species or genus level using *pplacer* [21]. The study identified a total of 155 unique microbial taxa.

### Classifier Details

We implemented a GP classifier in C++. Table 2 shows many of the parameters used by the genetic program. We used tournament selection with the worst model in the tournament group replaced by the child of the best. The GP created the child by either mutating the best model or crossing over the best model with the second best model in the tournament group. Due to high variability in the results of GP, we repeated the analysis ten times. The model with the highest training fitness was then selected for evaluation with the testing dataset.

**Table 2:** This table lists the parameter values used by the GP classifier.

Parameter	Value
Population size	15000
Tournament group size	4
Cross-over probability	0.2
Total generations	300
Mutation probability	1
Available node functions	addition, subtraction, protected division, multiplication,if/then/else, sine, cosine, logical AND, logical OR, maximum,minimum, log

The fitness of each model was calculated using two steps. The first step calculated a cutoff value for the classifier results. To calculate this value the classifier results were averaged for BV+ and BV− training cases separately. The cutoff value is the average of these two numbers. Values from the model that fell on or above this cutoff were considered BV+ classifications and values below this cutoff were considered BV− classifications. In order to generate the ROC plots shown in Figure 2, the fitness value of the BV+ training cases was multiplied by a constant that varied between 0 and 20. This constant allowed us to vary the value of classifying BV+ vs. BV− samples. In the second step, the total number of incorrectly classified samples was added to the size of the classification model multiplied by a small constant. This constant penalized larger models. The fitness was then minimized over the course of the program.

In order to identify features important to the GP models, we varied the values for each feature individually in every sample. We then determined whether varying the feature value changed the classification of the sample. This resulted in two summary values for each feature; the number of GP models in which varying the feature resulted in a different classification for at least one sample, and the number of samples in each model which changed classification due to changing the value of the feature. We rescaled these summary values to between 0 and 1 and added them together in order to obtain a single value describing the importance of each feature.

In order to implement the random forest classifiers we used the R package *randomForest* [22]. We used the *randomForest* function with default parameters to generate the classification model. To determine feature importance, we ranked the features by the increase in node purity. This is a measure of how much each feature increases the separation of the samples into BV+ and BV− categories for each classification tree. The increase in node purity was then averaged over all trees in the forest to obtain the total importance of each feature to the classification model.

To build a logistic model with linear regression, we used a maximum likelihood method implemented in the R package *glmnet* [18]. We ran the analysis using default parameters with a binomial response type. To determine feature importance, we ranked the features by the magnitude of the mean coefficient across the cross validation replicates divided by the standard deviation.

### Microbial Correlation Reduction

In order to reduce the number of parameters and to increase the interpretability of our results, we collapsed highly correlated microbes into groups. We calculated pairwise correlations on microbial relative abundances using *sparCC* [23]. We converted the correlations into dissimilarities by subtracting the magnitude of the correlation from one. We then used average hierarchical clustering and a dynamic tree-cutting algorithm to break the microbes into correlation groups. To do this we used the function *cutreeDynamic* from the R package *dynamicTreeCut* [24] with a 0.9 cut height and a three taxa minimum group size. This cut height was chosen to account for nearly all of the correlation present between microbes (Figure S1). Further analysis of the correlated groups is shown in Figure S2, which shows the mean cluster silhouette widths for varying cut heights. Uncorrelated microbes were left as individuals. A single feature in the dataset represented each correlated microbe group.

### Cross-validation and Accuracy Determination

In order to avoid model over-fitting, we used ten-fold cross validation [25]. Cross validation detects over fitting and indicates how well the model is expected to perform with new data. We randomly broke the data into ten different parts. We used nine of these parts to train the model and the remaining part to test the performance of the model. We repeated this nine other times, using each of the ten parts as the testing data. We then averaged the accuracy of the model in classifying the testing samples over each of the 10 datasets to obtain a measure for the accuracy of each machine-learning technique.

## Supporting Information

Figure S1 The complete clustering dendrogram for correlated microbes. This figure shows the complete dendrogram resulting from average hierarchal clustering of microbial correlations. The vertical red line shows the 0.9 cutoff used to define correlated microbe groups. As can be seen, this cutoff accounts for most of the correlation between microbes.
(EPS)

Figure S2 The average silhouette width of correlated microbe groups. This figure shows the mean silhouette width for correlated microbe groups at varying cutoff levels. We converted the *sparCC* correlations between microbial taxa to distances by subtracting the absolute value of the correlation from one. We then clustered the taxa using average hierarchal clustering. For each cutoff level, we defined correlated groups using the *cutreeDynamic* tree-pruning algorithm.
(EPS)
